# Sublethal Effects of Ionic and Nanogold on the Nematode* Caenorhabditis elegans*

**DOI:** 10.1155/2018/6218193

**Published:** 2018-11-01

**Authors:** Suanne Bosch, Tarryn Lee Botha, Anine Jordaan, Mark Maboeta, Victor Wepener

**Affiliations:** ^1^Water Research Group, Unit for Environmental Sciences and Management, Potchefstroom Campus, North-West University, Private Bag X6001, Potchefstroom, 2520, South Africa; ^2^Laboratory for Electron Microscopy, Potchefstroom Campus, North-West University, Private Bag X6001, Potchefstroom, 2520, South Africa; ^3^Unit for Environmental Sciences and Management, Potchefstroom Campus, North-West University, Private Bag X6001, Potchefstroom, 2520, South Africa

## Abstract

The nematode* Caenorhabditis elegans *is used as an ecotoxicological model species in both aqueous medium and solid substrates. It is easy and of low cost to maintain in the laboratory and it produces hundreds of offspring within a short period of time. It also has a small body size (1 mm), making it possible for* in vivo* assays to be conducted in 12-well plates. Engineered nanomaterials (ENMs) are a class of emerging pollutants. Nanogold (nAu) is used in many consumer products and* in vivo* drug delivery. These materials can be released into the aquatic environment during production or discarding of consumer products. As nAu is insoluble in water, the sediment would become the final depository for the materials. It has become increasingly important to use sediment dwelling organisms to screen for possible toxicity of these ENMs. In this study* C. elegans* was exposed to a range of concentrations of nAu and ionic gold in M9-media, acting as a substitute for pore water. After 96-hour growth, fertility and reproduction were determined. Internal structure damage and internalisation of particles in* C. elegans* were determined by using SEM and CytoViva® Darkfield Imaging. From these images the nanomaterials are distributed around the oocytes in the reproductive organs, as well as the pharynx. Results obtained indicate that nAu affects reproduction more than growth due to internal gonad damage, albeit at very high exposure concentrations, indicating no toxicity at environmentally relevant concentrations. Ionic Au is more toxic than nAu and effects fertility and reproduction due to ion release. These results give more information regarding the toxicity and* in vivo* uptake of nAu and form part of an environmental risk assessment of ENMs.

## 1. Introduction

Nanotechnology and the production of nanomaterials are growing fields of science and technology. Nanotechnology is broadly defined as the manipulation of matter at the nanoscale to produce new materials that have novel properties and functions [1]. Once the material that incorporates ENMs (engineered nanomaterials) starts becoming commercially produced, nanomaterials can be released into the environment during the production of the material or in succeeding activities like the packaging, transport, and storage of the product [2, 3]. Engineered nanomaterials can be released into the environment as waste or industrial pollution [4].

Nanogold (nAu) is used in many consumer products such as toothpastes, antiaging creams, masks, and* in vitro* drug delivery [5]. As the use and production of these particles increase, subsequently their potential risks to the environment intensify. Long-term responsible development of nanotechnologies depends heavily on better understanding of the fate and behaviour of ENMs in the environment. Currently there is limited information available on the effect of nanomaterials in terrestrial environments and even less so in pore water [6]. This can be achieved by increasing our current knowledge base on ENMs and the effect ENMs have on sediment-dwelling organisms. When released into water ENMs may settle down in sediment, sorb to sediment particles, or become transformed through biotic and abiotic processes [7]. The toxicity of metals in sediments has been shown to be predicted best by the pore-water concentration [8]. The bioavailability of metals in sediments is controlled by pore water and particle uptake by the organism, as the digestive fluids of benthic organisms cause particle-bound metals to become solubilized [9], a phenomenon which can be applied to metal nanomaterial studies via comparison.

Nematodes play an important role in benthic food webs [9]. They inhabit the interstitial water between organic and inorganic particles of sediment. The nematode* Caenorhabditis elegans* has become a widely used whole animal model for toxicology studies due to its dominance in the natural environment, easy culture maintenance within a laboratory, and known genetic and molecular knowledge. The nematode feeds on bacteria and other small particles by taking up liquid with suspended particles and then rejecting the liquid while keeping the particles in the pharynx [10]. This nematode also exhibits 60 – 80 % genome homology to humans and shares many biological traits, in terms of physiology and metabolism [11]. The intestine of this organism is very similar to that of higher animals, when looking specifically at intestinal cell polarity, the presence of cell junctions, and the presence of microvilli that form the brush border of the intestine [12]. Furthermore, the transport mechanisms of biomolecules through biological barriers are very well developed, similar to those of higher organisms [13]. All these aspects contribute to* C. elegans* being used as a promising tool to evaluate* in vivo* nanomaterial toxicity before moving to higher organisms, or even completely removing the need to test on higher organisms such as mammals. Therefore, the aim of this study was to evaluate the uptake and toxicity of both ionic and nAu in the nematode* C. elegans*.

## 2. Materials and Methods

### 2.1. *Caenorhabditis elegans* Maintenance

The* C. elegans* (strain N2) culture was obtained from the* Caenorhabditis* Genetics Centre at the University of Minnesota, MN, USA. The organisms were maintained in stocks of dauer larvae on nematode growth (NG) agar with* Escherichia coli* OP50 as food according to ISO standard procedures [14]. Stock cultures were kept in temperature incubators at 20 ± 2°C.

When synchronous nematodes were needed for a test, dauer larvae were transferred to an NG agar plate with a fresh lawn of* E. coli* OP50. After a period of three days at 20°C many gravid hermaphrodites, as well as L1 and L2 stage nematodes (juveniles), were present on the plate. According to ISO testing the first larval stage is used to start the test. To obtain nematodes synchronized to this life stage, the plates were first rinsed with M9-medium. This suspension, which contained nematodes of all stages, was then filtered through a 10-micron mesh size nylon net (Instrument Makers, North West University, Potchefstroom) and subsequently through a 5-micron mesh size net to retain larger juveniles and adults. The filtered suspension contained only first-stage juveniles (L1).

### 2.2. Nanogold Preparation and Characterization

#### 2.2.1. Nanomaterial Stock

The nAu stock solutions were prepared and supplied by MINTEK, a science council in South Africa. The stock solution was made up of 14 ± 2 nm nAu with product code TMU14G and batch numbers (20130304FKP49b; 20130308FKP52; 20140905BM001). The stocks were prepared by standard citrate reduction techniques and were sterilised using the filtration method [15, 16]. The ionic gold solution in the form of chloroauric acid (batch number 24855362) was purchased from Sigma-Aldrich. Stock solutions of 100 mg/L suspensions nAu and 2 mg Au/L ionic gold, respectively, were made in RO water (MilliQ Simplicity® UV), sonicated for an hour at 25°C (Scientech, Ultrasonic Cleaner), and maintained by storage at room temperature in darkness.

#### 2.2.2. Characterization

Before the initiation of the toxicity and growth inhibition experiments, nAu stocks were characterized in both RO water and media. The physicochemical properties that were determined were temperature, pH, and electrical conductivity, and total dissolved solids (TDS) were also measured for each concentration examined using an ExStik® II (Extech® Instruments, Taiwan), made up in M9-media. The characterization properties that were determined included zeta potential (surface charge), particle size, and shape. Transmission electron microscopy (TEM) was used to examine the particle shape, size, and aggregation patterns. Twenty microliters of nanomaterial suspensions were pipetted on and left to dry onto a 400-mesh carbon-coated copper grid and imaged with a FEI Tecnai G2 TEM at 200 kV. The excess media was removed using a filter paper by touching only the edge of the droplet and the grid was allowed to dry before examination [17]. Hydrodynamic size distribution as well as zeta potential, of all nAu concentrations in M9-media, was measured by using Dynamic Light Scattering (Malvern Zetasizer Nano Series, NanoZS).

Dissolution was measured by placing a known concentration of nAu suspension in a dialysis membrane within a beaker with simulated fluid. Particles can then move over the membrane and a concentration membrane develops over time when free ions can migrate to the simulated fluid in the beaker. This fluid was analysed for Au using inductively coupled plasma mass spectrometry (ICP-MS) to determine the degree of dissolution [18].

#### 2.2.3. Exposure Medium

According to the ISO 10872 (2010) guideline M9-medium (Table 1) was used as a medium for the nematode toxicity test providing analogous test conditions [19]. To make up the required exposure concentrations, relevant volumes of the sonicated stocks were added to the M9-media in each individual test well of a 24-well plate, in volumes of 1 mL/well (0.5 mL M9-nanostock and 0.5 mL* E. coli*).

### 2.3. Toxicity Tests

#### 2.3.1. Escherichia coli Preparation

A suspension of* E. coli* was prepared prior to the toxicity assay by inoculating 250 mL of sterilized Luria Broth [14] with 20 *μ*L of* E. coli *OP50. This culture was set on a shaker incubator at 37°C and 150 rpm for 17 h. The culture was then transferred into 250 mL polypropylene centrifuge tubes and spun at 2000 xg (Eppendorf Centrifuge 5430, Hamburg, Germany) for 20 min. The supernatant was decanted, and the bacteria pellets were resuspended in 50 mL of M9. This was repeated two more times. This suspension was then diluted in M9 and turbidity was measured using a Spectroquant (Pharo 300, Merck Millipore, Germiston, South Africa) and diluted to 1000 ± 50 Forzamin Attenuating Units (FAU; according to ISO). Cholesterol stock of 0.01% of the total bacterial solution volume (prepared according to ISO 10872) was added and shaken to mix properly. Five hundred microliters of this bacterial solution were then added to each well immediately at the start of the test as described below.

Benzylcetyldimethylammonium chloride monohydrate (BAC-C16) is used as a general positive control, to ensure that the condition and sensitivity of the organisms have not changed. The positive control was tested in parallel with each test, using a concentration near the EC_50_ of growth. The EC value was determined by exposing the nematodes to a range of concentrations between 7 mg/L and 36 mg/L BAC-C16 in water and determining growth inhibition as described below. The EC_50_ for BAC-C16 was determined as 15 mg/L which was the concentration used within this study.

#### 2.3.2. Toxicity Assessment

At the start of the test, 10 first-stage juvenile nematodes (J1) were transferred to each test well. The mean initial body length of the test organisms was determined using a Nikon H600L light microscope by measuring total length for 30 randomly selected J1 nematodes.

The toxicity tests were carried out according to the standard ISO 10872 (2010) protocol. Briefly, ten individuals were transferred to each well in a 24-well plate, in four replicates (N=40), using a micropipette. The exposure period lasted 96 h in darkness at 20 ± 2°C. For each test replicate, 500 *µ*L aliquot of both stock solutions at 2 times the required test concentration was diluted with a further 500 *µ*L of* E. coli* OP50 stock (1000 ± 50 FAU) to yield the desired test concentration in a 1 mL volume and the appropriate food density. The exposure concentrations for nAu exposure ranged between 1 and 100 mg/L, while ionic Au concentrations ranged between 0.005 and 2 mg/L as Au per litre.

The aspects examined were characterization of the nAu in the media. The biodistribution and uptake of Au within the nematodes, internal structure damage, and whole organism responses were also assessed as follows.

### 2.4. Exposure Assessment

#### 2.4.1. Biodistribution and Uptake of Internalized Gold

*CytoViva® Dark Field Imaging*. The biodistribution of nAu was examined by dispersing the transparent nematodes on a glass microscope slide after exposure and heat killing. Only the highest concentrations (100 mg/L nAu and 2 mg/L ionic Au) of each material were used to better our ability to detect internalization. The nematodes were not stained with Rose Bengal solution, preserved, or washed as to not remove any particles. Cryopreserve gel (Tissue-Tek® OCT™ Compound) was used to mount exposed nematodes onto a microscope slide and stored at 4°C until processing using CytoViva® 150 Unit integrated onto an Olympus BX43 microscope. Slides were carefully covered with a cover slip prior to imaging. Images of the exposure media and* C. elegans* were captured using the Dag excel X16 camera and DAGE Exponent software at 10- and 60-fold magnification. A Zeiss Celldiscoverer® microscope was also used to present images of the whole organisms, analysed using Zen blue software.

#### 2.4.2. Determination of Internal Structure Damage

*Scanning Electron Microscopy*. After the exposure period, live adult nematodes were collected using a micropipette; they were placed in TODD's fixative (Todd, 1986) for 2 minutes. Once immobile they were cut in half using a dissection blade under a dissecting microscope (Nikon SMZ 1500) at 11.25-fold magnification. Samples were left in Todd's fixative overnight at 4°C. Organisms were then mounted in agar (15 g/L bacteriological agar); small cubes with the samples inside were cut out and placed in fixative overnight at 4°C. These samples were then washed three times for 15 min in 0.05 M cacodylate buffer. Postfixation was done in 1 % Osmium tetroxide (made up in cacodylate buffer) for 1 hour. Dehydration of the material was done in an increasing ethanol series (50 %, 70 %, 90 %, 100 %, and 100 %) for 15 min. Ethanol was then replaced with 100 % LR White resin for 15 min and then replaced with fresh LR White resin twice for 45 min. The samples were then imbedded in fresh 100 % resin into gelatine capsules and placed at 65°C overnight. Capsulated samples were then trimmed using a Minora® blade and then ultramicrotomed using a diamond knife; the trimmed capsule surface was coated in carbon and imaged using a FEI quanta 250 FEG scanning electron microscope at 200 kV. Energy Dispersive Spectrometry (EDS) measurements were also taken of each image to identify any Au that may have been internalised.

### 2.5. Effects Assessment

#### 2.5.1. Growth, Reproduction, and Fertility

Following the 96 h exposure period, the samples were mixed with 0.5 mL of an aqueous solution of Rose Bengal (0.3 g/L) per well to stain the nematodes for easier counting. The test was stopped by heat killing the nematodes in an oven for 15 min at 80°C and finally stored at 4°C until further analysis. Nematodes were recovered and counted by transferring the aqueous medium from the test wells into a Petri dish using a Pasteur pipette. The number of adult male nematodes was noted per exposure replicate since they are unable to reproduce.

The reproduction was evaluated by counting the number of juvenile offspring under a dissecting microscope (Nikon SMZ 1500 with a Nikon DS-Fi one camera and ImageJ software) at 7-fold magnification. To determine offspring per test organism (*x*_*o*_) the following equation was used:

(1) $$\begin{array}{c} x_{o}=\frac{x_{j}}{x_{i}}-x_{m} \end{array}$$

Where

 
*x*_j_ is the number of juveniles observed in each sample;
 
*x*_i_ is the number of introduced organisms in each sample;
 
*x*_m_ is the number of males observed in each sample.

Length was determined by measuring the body length at 40-fold magnification using a light microscope (Nikon H600L, Nikon DS-Fi one camera and ImageJ software). Growth (*x*_g_) was then calculated by the following equation:

(2) $$\begin{array}{c} x_{g}=\bar{x_{l2}}-\bar{x_{l1}} \end{array}$$

Where

 
$\bar{x_{l2}}$ is the length of the organisms after exposure;
 
$\bar{x_{l1}}$ is the length of the 30 juveniles before exposure.

Fertility was measured by indicating the percentage of recovered hermaphrodites that were gravid in each replicate [14]. The inhibition of a test parameter,* x*_t_, is expressed as a percentage relative to the control, as given here:

(3) $$\begin{array}{c} x_{t}=\left(\;100-\frac{\bar{x_{s}}}{\bar{x_{c}}}\;\right)\times 100 \end{array}$$

Where

 
$\bar{x_{s}}$ is the mean of the parameter in a sample;
 
$\bar{x_{l1}}$ is the mean of the parameter in the control.

### 2.6. Statistical Analysis

The dose-response function, namely, effect concentration with x % of population affected (ECx values), were calculated using nonlinear regression analysis in ToxRat®. To test for normal distribution, Shapiro-Wilk's test was used and the Levene test was used to test for variance in homogeneity. One-way Analysis of Variance was performed to calculate the statistical differences (p < 0.05) between the different concentrations of the nAu, as well as any significant differences between the control and the nanomaterial. Kruskal-Wallis test was performed to calculate the statistical differences for all the data that were not normally distributed.

## 3. Results

### 3.1. Characterization

Physicochemical parameters of M9-medium containing the 10 different exposure concentrations were measured at the beginning of the tests. The physicochemical parameters in the stock solution (100 mg/L) were analysed in RO water. Temperature ranged between 20 and 22°C. The pH of the control media was around 7.7, conductivity was 15.52 mS/cm, and TDS was 10.94 g/L.

**Figure 1:**
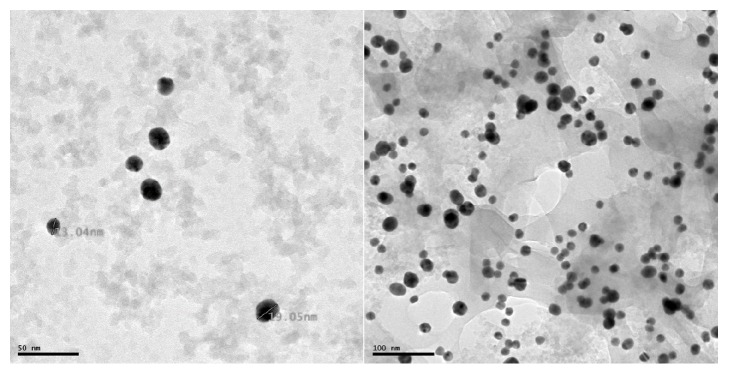
Transmission electron microscopy images of nanogold particles in citrate buffer 1 g/L.

Nanogold particles were stable in the citrate stock solution and maintained a range of 14 ± 2 nm within the stock when checked using TEM (Figure 1). However, once in M9-media suspensions particle sizes ranged between 132 and 667 nm (Table 2). The lowest concentration showed the largest particle size, but agglomeration does occur at the highest concentration, causing the largest particles to sediment out thereby avoiding detection. Dissolution of ions from the particle was less than 5 %. The conductivity in the M9-media ranged between 10 and 15.4 mS/cm for the nAu exposures and 8 and 15.9 for the ionic Au exposures. The lowest conductivity is that of ionic Au 2 mg/L and nAu 100 mg/L, and the highest is that of ionic Au 0.005 mg/L. As the concentrations increase the TDS and conductivity decrease and pH was in the normal range. The pH during the nAu and ionic Au exposures varied between 7.15 and 7.32.

### 3.2. Biodistribution and Uptake

**Figure 2:**
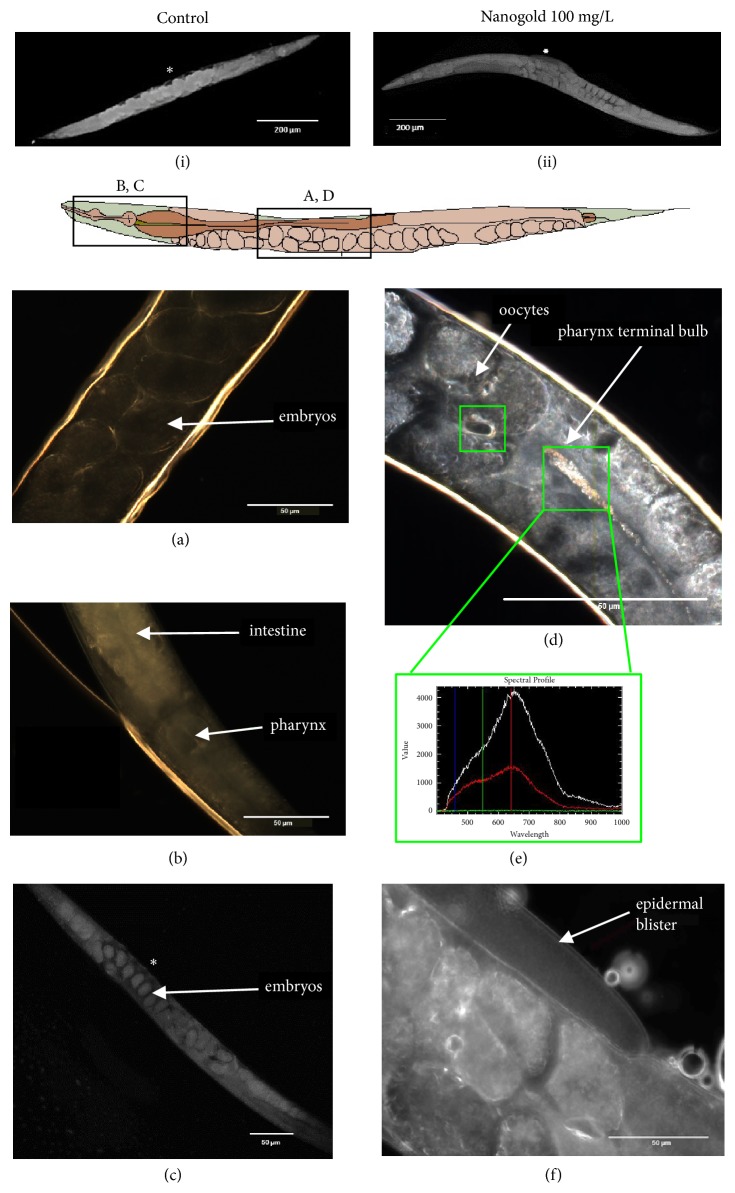
CELLDISCOVERER 7 images (i, ii, c) and CytoViva® Darkfield images of control nematodes (a and b) and a nematode exposed to the highest concentration nanogold (ii, d, f). The image also indicates the section of the organism where images are located as well as the spectral profile (e), indicating gold present in the pharynx and oocytes (d). Epidermal blisters were also observed around the vulva area (f). Asterisk indicates the vulva.

From images collected using the Zeiss CELLDISCOVERER 7® (Figures 2(i) and 2(ii)) it was evident that blisters formed around the vulva.* CytoViva® *was then used to further observe these blisters and to confirm internalisation of particles. Particles can be seen in the terminal bulb of the pharynx and in the oocytes in the anterior branch of the gonad (Figure 2(d)). The hyperspectral profile indicates background noise in green, tissue in red, and gold in white (Figure 2(e)). This confirms the presence of gold particles within the pharynx and around anterior oocytes (Figure 2(d)), compared to the control (Figures 2(a)–2(c)). Thus, small amounts of nonaggregated particles that are smaller in size have the potential to move from the pharynx where they are ingested to other internal structures such as the oocytes. It is however unclear yet as of if the particles enter the oocytes. Epidermal blisters (separation of cuticle layers) can also be seen (Figure 2(f)), which was only observed in organisms exposed to nAu and was only present around the vulva. In Figure 2(ii) it can be seen that the blisters only occurred at the vulva, compared to the control (Figure 2(i)).

### 3.3. Determination of Internal Structure Damage

**Figure 3:**
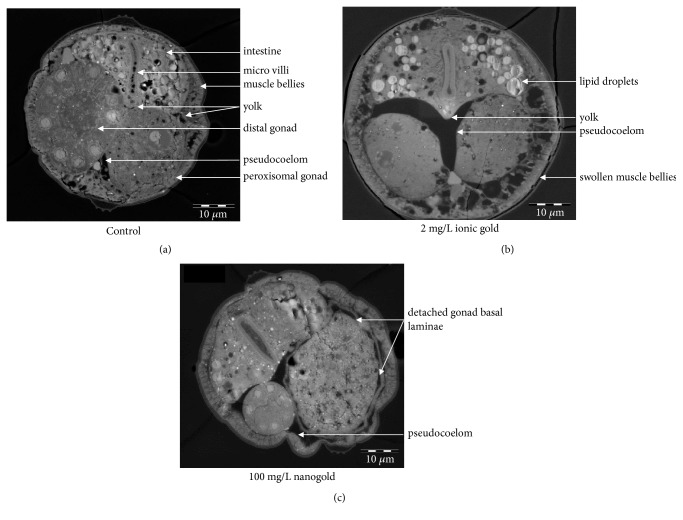
Scanning electron micrograph images of cross-sections of* C. elegans *exposed to the highest concentrations of ionic (b), nanogold (c), and control (a).

Further analysis was done using SEM back scatter images to determine internal changes. The SEM back scatter images (Figure 3) indicated swollen muscle bellies on the ventral side of ionic and nAu exposed organisms; this could decrease locomotion ability and eventually feeding problems in the organisms. From Figure 3(b) it is evident that there are more lipid droplets present in the intestinal area of nematodes exposed to ionic Au.

Furthermore, there is an obvious enlargement of the pseudocoelom between the distal and peroxisomal gonads (Figure 3(b)). The peroxisomal gonad basal laminae (BL) of nematodes exposed to nAu also seem damaged and deformed as seen by the shape and detachment of the BL from the gonad (Figure 3(c)), and it is unclear if any yolk is reaching the developing oocytes in both cases due to the lack of yolk in the pseudocoelom. This may put reproductive strain on the organisms. Generally, germline development suggests the animals were thriving under both conditions with regard to growth and development. All images were compared to the control (Figure 3(a)), as well as resources from WormAtlas [20]. Gonad size differences observed are due to the locality and orientation of the specific cross-section, not due to adverse effects from exposure.

### 3.4. Effects Assessment

Growth, fertility, and reproduction were used to calculate EC_x_ values for all the materials. For some of the materials EC_50_ values could not be calculated and therefore EC_10_ and EC_20_ values with upper and lower confidence limits (CL) are also reported. The NOEC and LOEC values were also calculated.

Comparing nAu to ionic gold, it is evident that nAu toxicity based on EC_10_ is much lower for reproduction and there are no significant differences in growth (Table 3). Reproduction was the most sensitive endpoint since EC values could be calculated for 10, 20, and 50%. The NOEC and LOEC values for nAu growth are greater than 100 mg/L. However, the ionic Au had calculable NOEC and LOEC values.

**Figure 4:**
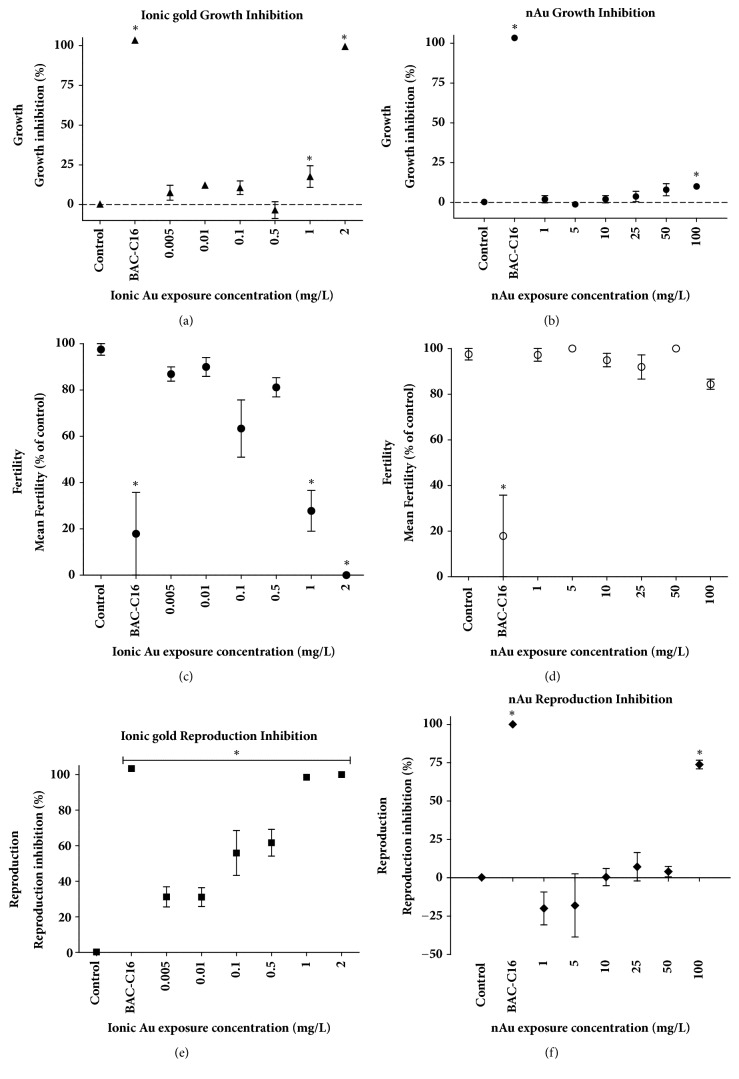
Growth inhibition (% inhibition), fertility (% of control), and reproduction (% inhibition) of nematodes exposed to ionic and nanogold. The asterisks indicate statistical difference (p < 0.05) from the control.

Ionic Au has much higher toxicity compared to the nAu exposure concentrations. Nanogold showed a slight dose-dependent increase in growth inhibition with only the 100 mg/L exposure being statistically higher than the control (Figure 4(b)). Ionic Au showed a bimodal response pattern, increasing from 0.005 to 0.1 mg/L, then decreasing to 0.5 mg/L and increasing rapidly to 2 mg/L exposure (Figure 4(a)). Only the 2 mg/L growth inhibition differed statistically from the control. The growth of the general positive control (BAC-C16) was statistically different from the control in each exposure. Nanogold showed no statistical difference in fertility compared to the control (Figure 4(d)). Nematodes exposed to ionic Au showed a bimodal response with a dose-dependent decrease between 0.5 mg/L and 2 mg/L exposure concentrations (Figure 4(c)). Only 1 mg/L and 2 mg/L had a fertility value statistically lower than the control. Nematodes exposed to nAu presented a slight dose-dependent increase in reproduction inhibition; however, none of the concentrations had a reproduction statistically different from the control (Figure 4(f)). There was a reproduction stimulation at 1 and 5 mg/L concentrations of nAu, but not significant. The reproduction of ionic Au exposed nematodes showed a more notable dose-dependent increase, with all concentrations statistically different from the control (Figure 4(e)).

## 4. Discussion

In this study there was a reduction in conductivity of the spiked exposure media at the highest concentrations tested. The lower the conductivity is, the fewer the ions are present [21]. As the concentrations increased, the ions and TDS in the medium decreased causing lower conductivity. This can be attributed to the agglomeration of particles at higher concentrations in M9-media [22, 23].

Ionic Au exposures had significantly higher TDS and conductivity values than nAu. This means more metal ions were present in the ionic solution; thus, toxicity differences between ionic gold and nAu could be attributed to the free ions of ionic Au. It is also important to note that pH could have an effect on ion release and toxicity of ENM [24]. Even though the pH of the media stayed relatively neutral over all concentrations,* C. elegans* has an intestinal pH ranging and oscillating between 3.5 and 6 [24]. According to the literature when pH is low particles can agglomerate or be acidified into their ionic form; this could happen within the intestine and not necessarily in the media [25, 26].

Ivask* et al*. [27] found that larger particles ranging between 20 and 80 nm had higher dissolution rates than smaller particles (10 nm). However, smaller particles were able to interact with cell membranes more readily [27]. In this study the mean nAu size range was 132 nm, with less than 5 % dissolution. The initial particle size of 14 nm that was used agglomerated in the exposure media, thereby resulting in larger particles. A concentration-dependent agglomeration pattern was observed, with the highest concentration forming the smallest aggregates. The smaller agglomerates could be the cause of toxicity observed at this concentration. A study by Roh* et al.* [28] found that smaller CeO_2_ ENMs were more toxic to* C. elegans*, and toxicity was observed at much lower concentrations (1 mg/L) than this study.

Particles were visualized using CytoViva® Darkfield imaging, but not by using SEM, as no metal was detected within organs by EDS (data not shown). Nanogold was identified in the pharynx and oocytes. The presence of particles in the pharynx is likely due to feeding behaviour; the nematode ingests particles while feeding as they suck up food through the buccal cavity. The particles end up in the pharynx and intestine. A study has confirmed translocation of silica NPs and polystyrene NPs from the pharynx to the lumen and early embryos/oocytes depending on their composition [29]. A study by Taylor* et al*. [30] observed nAu particles internalized in great numbers into the oocytes as observed by confocal microscopy. Thus, it is possible for the particles to cross internal barriers from the pharynx to the adjacent developing oocytes. However, nAu particles were observed not to influence oocyte function at 50 mg/L and below [30]. Similar studies using nAu showed that they are not able to cross intestinal and dermal barriers [11]; in this study we have used particles in much smaller ranges than the previous study; thus it is likely smaller particles can have the ability to cross these barriers.

Scanning electron micrographs did, however confirm more lipid droplets present in the intestine of nematodes exposed to the highest concentration of ionic Au in this study. Lipid droplets are a class of eukaryotic cell organelles for storage of neutral fat such as triacylglycerol and cholesterol ester [31]. Increased presence and size of lipid droplets can be caused by genetic defects in a peroxisomal beta-oxidation pathway in* C. elegans* [31]. Results of Zhang et al. [31] suggest that lipid droplet size in* C. elegans* is dynamic and is linked closely to storage, mobilization, and peroxisomal catabolism of fat. This means that enlarged droplets observed within the intestine after exposure to ionic Au can be due to disruption of the peroxisomal beta-oxidation pathway. This can be caused by oxidative stress and causes a disruption in the pathway that breaks down lipids for energy.

The pseudocoelom, which is the body cavity of the nematode, provides the turgor-hydrostatic pressure for the animal as a whole, functions as a lubricant between tissues, and provides a medium for intercellular signaling and nutrient transport [20]. The coelomocytes cells that are found within the pseudocoelom perform an immune surveillance function for the animal. They can recognize substances, viruses, or invading bacteria that do not belong inside the animal and degrade them. Thus, the reason for the enlarged pseudocoelom (more pseudocoelomic fluid) could possibly be due to an immune response following exposure to both ionic and nAu, but this is not confirmed.

Micrographs did not show any yolk in the pseudocoelom and very little yolk present in the intestine of ionic and nAu exposures. This could be an indication that yolk did not reach the oocytes and the consequent inhibition of reproduction that was observed at the highest concentrations of both ionic and nAu. In* C. elegans*, yolk is secreted from the intestine, where it is synthesized, into the pseudocoelomic space as free-floating granules and is ultimately transported to the reproductive tract. When reproductive life has ended, excess yolk begins to collect within the body cavity to form huge islands of yolk material [32]. From the micrographs a noticeable damage to the gonad BL of nematodes exposed to nAu was also seen by the shape and detachment of the gonad BL from the peroxisomal gonad itself. These yolk granules need to move through the BL and gonad sheath pores to the surface of the oocyte, where they are taken up into vesicles within the growing oocytes to successfully nourish them [33, 34]. This can explain the inhibition of reproduction without influencing the fertility; namely, the oocytes are formed but there are insufficient nutrients in the yolk. The Darkfield CytoViva® images also showed what seems like epidermal blisters in the vulva region of the nematode after exposure to the highest concentration of nAu, as observed in* C. elegans* mutants that have reduced tetraspanin protein function [35]. This could also affect egg release in the nematode and is caused by either mutation in at least six genes, bli-1 to bli-6, or a deficiency of tetraspanin protein, which is essential for epidermal integrity.

Effect concentration values indicated that reproduction was a more sensitive endpoint than growth and fertility. These EC values for reproduction also indicate that ionic Au (EC_10_= 0.002 mg/L) was more toxic than nAu (EC_10_= 68.51 mg/L). In both exposures growth was affected at much higher concentrations than reproduction; this could be due to different mechanisms of toxicity; as previously mentioned, these mechanisms could include damage to the gonads, cellular uptake, and cellular membranes by the formation of reactive oxygen species, as well as oxidative stress [36]. Studies such as those of Kim* et al.* [37] and Gonzalez-Moragas* et al.* [11] also found that reproduction could be affected by nAu particle exposure, but both studies were carried out in agar or by feeding the nematodes with exposed* E. coli*, rather than in more comparable and natural media such as M9 media used in this study. Particles also differ in size when comparing studies. As previously mentioned particle size has an effect on toxicity; this can explain the slight difference in results. However, the fact that both our results and theirs indicate reproductive toxicity could be due to similar modes of toxicity such as oxidative stress for particles of different sizes [36].

Only ionic Au showed a significant effect on fertility. This reduced fertility capacity and growth inhibition by ionic Au can be explained as a part of compensatory or defence mechanism to reduce the toxicity induced by Au ions, since increased lipid droplets indicate a metabolomic pathway disruption that could be a compensation due to exposure [28]. Nanogold did have a significant effect on* C. elegans* growth at the highest concentration and this is similar to the results by Roh et al. [28] and Contreras* et al.* [38] that found no significant effects on growth of* C. elegans* after exposure to CeO_2_ ENMs and core-shell QDs (CdSe/ZnS), respectively. Ionic Au also showed toxicity to growth, fertility, and reproduction at much lower concentrations than its nanoequivalent, making it 100-fold more toxic. This is similar to the findings by Hsu* et al.* [39] following exposure of* C. elegans* to QDs.

## 5. Conclusions

The uptake and effects of nAu and its ionic salt were evaluated in this study using* C. elegans* as a model organism. Both substances caused growth inhibition in* C. elegans* at the highest exposure concentrations. Clear dose-dependent differences were also seen in reproduction following exposure to ionic and nAu. However significant differences in the dose-response curves of fertility were not observed. Ionic Au caused reduced reproduction and growth in* C. elegans*, but a low incidence of fertility deficiency was observed, thus indicating that* C. elegans* was affected by different mechanisms when comparing ionic and nanoforms. Overall bulk metal salts are considerably more toxic than their nanoequivalents. From this study, we can also deduce that nAu could have low bioavailability in the gut of nematodes as the biological effects are far less severe than the ionic salt. Based on CytoViva® images, it was found that nAu particles are both ingested and internalized by* C. elegans*. This was in contrast to the SEM images and EDS, which could not confirm the internalization. Particles within the pharynx and oocytes can impair growth, inhibiting nutrient uptake and egg laying deficiencies, respectively. Reproductive abnormalities support findings of internal reproductive structure damage and yolk deficiencies. The presence of epidermal blisters around the vulva can be the cause of reduced reproduction due to impaired egg release. Previous studies [11, 37] have indicated reproductive toxicity on* C. elegans* due to nAu, but none have observed growth inhibition, as well as epidermal blisters; this is a novel finding. Comparing ionic Au to nAu under more natural conditions is also important for comparative toxicity.

## Figures and Tables

**Table 1:** Prepared amounts of salts added to one litre RO water to prepare M9-media (ISO 10872, 2010).

**Component**	**Mass (grams)**
Na_2_HPO_4 (Sigma−Aldrich, batch number BCBQ4788V)_	6
KH_2_PO_4 (Rochelle Chemicals, batch number 210812PO)_	3
NaCl_ (Promark Chemicals, batch number 35626.5925)_	5
MgSO_4_ · 7H_2_O_ (Promark Chemicals, batch number 34115/4919)_	0.25

**Table 2:** Characterization of nAu in M9-media (mean ± SD).

**Nominal concentration nAu (mg/L)**	**Size distribution (nm)**	**Zeta potential (mV)**
**1**	667 ± 57.6	-12.7 ± 0.818

**5**	431 ± 15.9	-14.8 ± 1.14

**10**	326 ± 21.8	-15.4 ± 1.88

**25**	257 ± 1.31	-15.5 ± 1.54

**50**	212 ± 0.525	-13.2 ± 0.377

**100**	132 ± 1.77	-15 ± 0.865

**Table 3:** Effect concentration (mg/L) values of nanogold and its bulk chemical equivalent.

	**Au**	**Toxicity parameters with upper and lower confidence limits (mg/L) 95%-CL**							
		**EC**_**10**_		**EC**_**20**_		**EC**_**50**_		**NOEC**	**LOEC**
**Growth**	nAu	95.8^a^	209.8	n.d.	n.d.	n.d.	n.d.	n.d.	n.d.
			43.8		n.d.		n.d.		
	Ionic Au	0.97^a^	1.09	1.06	1.23	1.24	1.74	0.5	1
			0.87		0.90		0.87		

**Reproduction**	nAu	68.5	n.d.	91.9	n.d.	161.3	n.d.	>=100	>100
			n.d.		n.d.		n.d.		
	Ionic Au	0.002	0.074	0.007	0.20	0.06	3.19	n.d.	n.d.
			0		0		0.001		

(1) n.d.: not determined. (2) Values within the same column with the same superscript symbol (a) are statistically different (p < 0.05). This could only be determined for values with both upper and lower confidence limits.

## Data Availability

The data used to support the findings of this study are available from the corresponding author upon request.
